# Vascular Malformation and Common Keratinocytic Nevus of the Soft Type: Phacomatosis Pigmentovascularis Revisited

**DOI:** 10.1155/2014/437085

**Published:** 2014-11-23

**Authors:** André Laureano, Rodrigo Carvalho, Cristina Amaro, Isabel Freitas, Jorge Cardoso

**Affiliations:** Department of Dermatology and Venereology, Hospital de Curry Cabral, Centro Hospitalar de Lisboa Central, Rua da Beneficência, No. 8, 1069-166 Lisboa, Portugal

## Abstract

Phacomatosis pigmentovascularis is a rare syndrome characterized by the coexistence of a pigmented nevus and a cutaneous vascular malformation. We report a 5-year-old boy with all the typical findings of phacomatosis pigmentovascularis type Ia. Although its existence according to the traditional classification has been questioned, this case represents a very rare association of a capillary vascular malformation and a common keratinocytic nevus of the soft type.

## 1. Introduction

Phacomatosis pigmentovascularis (PPV) is a rare syndrome characterized by the coexistence of a pigmented nevus and a cutaneous vascular malformation. The diagnosis of such syndrome is primarily clinical and further classification is based on the type of pigmented nevus [1].

## 2. Case Presentation

**Figure 1:**
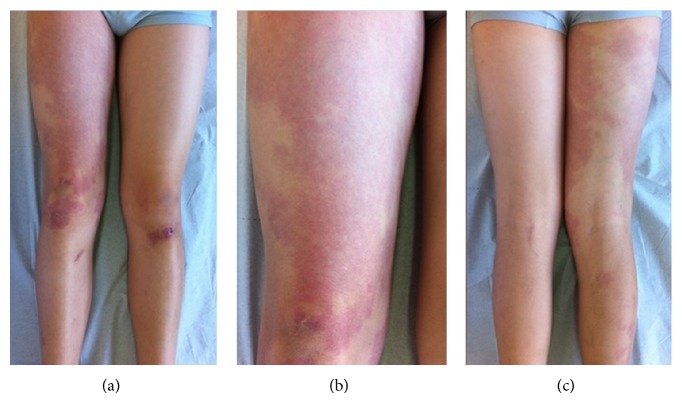
Multiple pink-red, irregularly shaped patches on the right lower limb (a)–(c).

**Figure 2:**
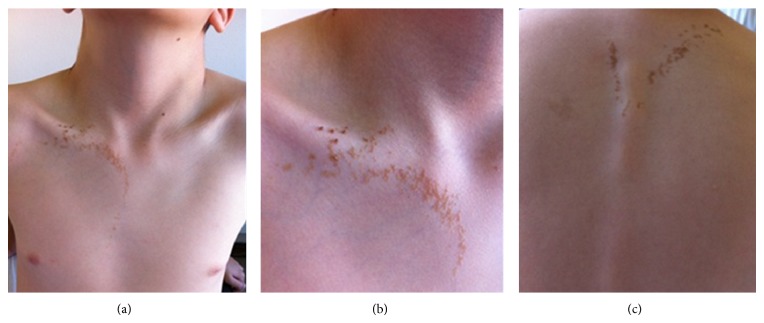
Linear brown verrucous plaques on the right side of the chest and upper dorsum (a)–(c).

**Figure 3:**
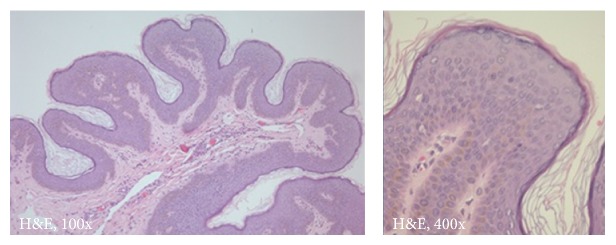
Histopathological examination revealed epidermal acanthosis, papillomatosis, and hyperkeratosis.

A 5-year-old boy presented to our clinic with asymptomatic, pink-red, irregularly shaped patches on the right lower limb (Figures 1(a)–1(c)) and also linear brown verrucous plaques on the right side of the chest (Figures 2(a)–2(c)). Both were seen since birth. The remaining examination was normal without further neurological or ocular abnormalities. Magnetic resonance angiography of the right lower limb was normal. Histopathological examination of the verrucous plaque showed acanthosis with epidermal papillomatosis and hyperkeratosis (Figure 3).

The combination of these findings favoured the diagnosis of a capillary vascular malformation (CVM), nevus flammeus type, and a common keratinocytic nevus of the soft type. Hence, typical findings of PPV type Ia according to the traditional classification were observed. No further progression was seen after 7 years of follow-up.

## 3. Discussion

PPV was first described by Ota in 1947. Since then around 245 cases have been reported, being mostly sporadic [1, 2]. Its pathogenesis is still poorly understood [3].

PPV was traditionally classified into five types according to the associated pigmented lesion. Each type was further divided if only cutaneous signs were present (subtype a) or if there were also associated extracutaneous signs (subtype b). The most commonly described was type IIb (about 45%), followed by type IIa (30%). Half of the patients have systemic involvement [1, 4].

Recently, Happle [5] proposed a revised classification which is simpler as it divides PPV into four groups: phacomatosis cesioflammea (coexistence of large blue macules asymmetrically arranged and an extensive nevus flammeus), phacomatosis spilorosea (macular nevus spilus coexisting with nevus roseus with a lighter hue than nevus flammeus), phacomatosis melanorosea (one or more large lateralized café-au-lait macules with nevus roseus), phacomatosis cesiomarmorata (large aberrant Mongolian spots and cutis marmorata telangiectatica congenita), and “phacomatosis cesioanemica” (blue nevus coexistent with nevus anemicus). Differentiation between cases with or without extracutaneous features was eliminated.

We reported a patient with all the typical findings of PPV type Ia which was previously described only in few reports. This type was also rejected in Happle's classification considering that a common keratinocytic nevus does not originate from pigment cells. In our case, a rare and now unclassifiable association between a CVM (nevus flammeus) and a common keratinocytic nevus of the soft type with a lateralized pattern was seen. This skin mosaic pattern cannot be explained by a nonallelic twin spotting or didymosis phenomenon, which is now considered as untenable and proven to be wrong [3, 6]. It is unlikely that PPV or other binary genodermatoses involving two different tissues might be explained by a loss of heterozygosity. In phacomatosis cesioflammea, nevus flammeus was caused by a heterozygous GNAQ mutation. The same was true in phacomatosis pigmentokeratotica because its two components were found to originate from one single pleiotropic HRAS mutation present in a heterozygous state, being now considered as an example of “pseudodidymosis” [7]. An arbitrary coincidence of this presentation cannot also be excluded.
